# Two decades of research capacity strengthening and reciprocal learning on sexual and reproductive health in East Africa – a point of (no) return

**DOI:** 10.1080/16549716.2024.2353957

**Published:** 2024-06-03

**Authors:** Caroline Frisendahl, Emelie Looft-Trägårdh, Amanda Cleeve, Susan Atuhairwe, Elin C. Larsson, Othman Kakaire, Herbert Kayiga, Annette Aronsson, Anne Kihara, Marleen Temmerman, Marie Klingberg Allvin, Josaphat Byamugisha, Kristina Gemzell Danielsson

**Affiliations:** a Department of Women’s and Children’s Health, Karolinska Institutet and Karolinska University Hospital, Stockholm, Sweden; b Department of Global Public Health, Karolinska Institutet, Stockholm, Sweden; c Department of Obstetrics and Gynaecology, Makerere University College of Health Sciences, Kampala, Uganda; d Department of Obstetrics and Gynecology, School of Medicine, University of Nairobi, Nairobi, Kenya; e Department of Obstetrics and Gynecology, Aga Khan University, Nairobi, Kenya; f Faculty of Medicine and Health Sciences, Ghent University, Ghent, Belgium

**Keywords:** Global health, SRHR, comprehensive abortion care, post abortion care, contraception, Swedish research funding, East Africa, research collaboration

## Abstract

As the world is facing challenges such as pandemics, climate change, conflicts, and changing political landscapes, the need to secure access to safe and high-quality abortion care is more urgent than ever. On 27th of June 2023, the Swedish government decided to cut funding resources available for developmental research, which has played a fundamental role in the advancement of sexual and reproductive health and rights (SRHR) globally, including abortion care. Withdrawal of this funding not only threatens the fulfilment of the United Nations sustainable development goals (SDGS) – target 3.7 on ensuring universal access to SRHR and target 5 on gender equality – but also jeopardises two decades of research capacity strengthening. In this article, we describe how the partnerships that we have built over the course of two decades have amounted to numerous publications, doctoral graduates, and important advancements within the field of SRHR in East Africa and beyond.

## Paper context

- **Main findings:** The two-decade long collaboration between Sweden and East Africa, funded by the Swedish government, has resulted in important partnerships, research findings, and advancements within sexual and reproductive health and rights in East Africa.
- **Added knowledge:** The Swedish government is now cutting funding for development research, which jeopardises the progress made so far.
- **Global health impact for policy and action:** Governments need to prioritise women’s sexual and reproductive health and rights.

## Background

Throughout history, women’s sexual and reproductive health and rights (SRHR) have not been prioritised by governments, nor in research agendas, with abortion being the most heavily debated and politicized SRHR issue [1]. While medical abortion is a highly effective and very safe procedure, unsafe abortion is one of the main causes of maternal deaths globally. Consequently, improving access to safe abortion, and access to modern contraceptives, is an efficient way to reduce maternal mortality and morbidity [2].

Abortion care is included on the WHO’s list of essential healthcare since 2020 [2], and while there has been advances in SRHR in recent years, the world has also experienced a significant backlash leading to restrictions on access to abortion care [3]. For instance, the reinstatement of the Mexico City Policy in the US in 2017 prohibited foreign non-governmental organisations from providing, referring, and advocating for or counselling on abortion as a method of family planning which resulted in decreased access to SRHR services in many low- and middle-income countries [4]. This was followed by the upheaval of Roe vs Wade in 2022, ending the constitutional right to abortion in the US, giving anti-choice movements, increasing ground to target countries outside of the US including East Africa. This backlash has already impeded progress towards the SDGs. To prevent that progress completely stalls, it is imperative that efforts, including advocacy and research, to ensuring universal access to safe abortion and contraception can proceed, without delays.

## Swedish government cuts funding to developmental research

The Swedish Research Council (VR) and the Swedish International Development Cooperation Agency (Sida) are research agencies funded by the Swedish government. In 2013, VR was ranked top 25 among the largest research funders globally [5]. On the 27th of June 2023, the Swedish Government announced that VR would no longer be offering grants for developmental research, taking 17 million USD off the table [6]. This decision, which was taken after the grant submission deadline, was made without any public discussion or a consultation with Swedish researchers. Approximately 250 researchers in Sweden with international collaborators, mainly in universities in Africa and Asia, were affected [6]. The decision sparked outrage amongst the research community, who in an open letter argued that cutting grants for developmental research seriously risks undermining progress made and the future of Swedish research to this regard [6]. These funding cuts coincide with dramatic reductions (>50%) in governmental funding for Sida for developmental research in 2023 and 2024 (from 980 to 43 million USD per year) and global cuts to Official Development Assistance and SRHR funding [7].

## Two decades of building partnerships, research capacity, and collaborations

In recent decades, numerous collaborations have emerged between universities in Sweden and East Africa, facilitated by research funding from the Swedish government. An example is the 21-year long capacity strengthening program between Karolinska Institutet (KI) in Sweden, and Makerere University in Uganda, supported by Sida. Through a sandwich model with double PhD degrees from KI and Makerere, a doctoral education program was established at Makerere University, enabling doctoral students to pursue a PhD degree in Uganda. By 2015, the collaboration had resulted in 60 PhD degrees with 82% of the PhD students from Uganda and over 500 peer-reviewed published articles, the majority featuring a Ugandan as either the first or last author [8]. The alumni networks created through the universities comprise hundreds of researchers and health workers from countries such as Uganda, Kenya, Rwanda, Somalia, and Congo. Furthermore, VR’s Developmental Research Grant has enabled long-standing partnerships between universities in Sweden, Uganda, and Kenya and contributed to research capacity strengthening in all these settings. VR grants have also enabled networking across Eastern and Southern Africa through a series of four workshops in comprehensive abortion care, organised in Kampala and Nairobi between 2016 and 2022 [9,10]. These workshops have promoted networking and supported the creation of new partnerships. Further, they have resulted in knowledge translation and dissemination and joint priority setting in terms of research, policy, and action [9,10].

Our collaboration between KI and universities in East Africa has proven to be mutually beneficial for the countries involved by enabling the exchange of resources, expertise, and scientific ideas and improvements in university education through reciprocal learning. Furthermore, they have resulted in high-quality evidence, outstanding academic achievements, and contributed to both national and international guidelines, policy, and practice reforms within the field of SRHR (Table 1). The long-term nature of our partnerships, enabled by Swedish governmental funding, has been crucial in these achievements and our efforts to strengthen capacity in research and clinical practice.

**Table 1.** Important advancements in the field of abortion care in East Africa spanning two decades.

• Safer abortions through improved access to misoprostol increase use of medical abortion and a decreased use of harmful methods.
• Improved access to PAC by task sharing and task shifting from physicians to midwives
• Increased contraceptive uptake through peer-counselling, youth friendly clinics, and interventions towards refugees
• Improved understanding and a decrease in contraceptive and abortion-related stigma through validated stigma scales and awareness
• Increased use of telemedicine for abortion care and contraceptive services

Abbreviations: PAC = Post Abortion Care.

## Advancements in SRHR in East Africa supported by Swedish governmental funding

The long-term collaboration described above have yielded substantial advancements for SRHR in East Africa, notably in Kenya and Uganda, and are described below.

## Contraceptive counselling and use

Our collaboration has generated evidence on emergency contraception in Uganda [11,12], revealing a lack of awareness surrounding fertility and emergency contraception among both healthcare providers and women of childbearing age. Further, the partnership has provided knowledge surrounding the safety, effectiveness, and acceptability of intrauterine devices (IUDs) among HIV-positive women [13]. Several studies have focused on postabortion contraceptive counselling and use. Research from Uganda has highlighted opportunities and challenges in provision of post-abortion contraception and how social norms impact who is counselled and how [14,15]. In Kenya, studies have shown the promises of integrating contraceptive counselling in postabortion care but also revealed persisting challenges in providing counselling that leads to initiation and continued use over time [16,17]. In Northern Uganda, Bakesiima et al. showed how contraceptive counselling by peers (trained adolescents) can increase contraceptive uptake among female adolescents in refugee settlements [18].

## The use of misoprostol and task-sharing to midwives

Access to the medicine misoprostol has increased during the last two decades in both Uganda and Kenya, along with a higher acceptance among healthcare providers and increased use of misoprostol in PAC, consequently decreasing the use of unsafe methods, such as sharp curettage [19]. Building on this, our collaboration has produced several research outputs. For instance, our research has shown that task sharing in the management of first and second trimester incomplete abortion using misoprostol is equally safe, effective, and acceptable to women. This evidence has contributed to improved access to safe PAC in Kenya and Uganda in recent years [20–22]. Further, it has contributed to the WHO recommendations on task sharing in first trimester PAC [2], with special significance for settings with strained healthcare systems and skewed distribution of health workers.

## The role of stigma in contraception and abortion

The collaboration has also researched societal and institutional stigma related to the use of contraception and abortion as major barriers in accessing SRHR services [23,24]. In Uganda, our research has revealed the negative impact of abortion stigma on abortion pathways and care seeking behaviours of young women [25] and how institutional stigma impedes quality of PAC provision [14]. Scales to identify and measure contraceptive and abortion-related stigma among adolescents have been developed and validated in Kenya [23]. Findings, using these scales, show that both the use of contraceptives and abortion is highly stigmatised among adolescents in Kenya, especially among male students [26]. These finding highlight the need for de-stigmatization efforts, engaging communities, and addressing stigma in training curricula and within healthcare institutions. Research on interventions that effectively reduce stigma are missing and yet the cuts to Swedish government funding limit our ability to advance this work.

## Telemedicine for providing abortion and contraceptive services

Our research has showed how the COVID-19 pandemic highlighted the need for further development and implementation of new telemedicine models for medical abortion [27]. Telemedicine provision for first trimester medical abortion provides a unique possibility for women and girls to safely self-manage part of, or the entire, abortion process and could significantly increase access to care [28,29]. The use of telemedicine in the contexts such as Kenya and Uganda need to be systematically tested and evaluated before scaling up implementation. Our applications for projects focusing on telemedicine for medical abortion in these contexts were immediately and effectively cancelled when the Swedish government cut funding for developmental research in 2023. We consider this a major missed opportunity to realise the SRHR of women and girls and to advance the agenda towards SDG3 and 5.

## Conclusions

Advancements in SRHR presented in this paper serve as important examples of how government funding for development research can build fruitful partnerships, strengthen research capacity, and progress the SDG agenda. The kind of mutually beneficial partnership described in this paper requires long-term investments and efforts. It is now at risk due to government cuts in research funding. To ensure that progress in realizing the SRHR of women and girls does not stagnate, efforts to safeguard universal access to good-quality care must not cease. SRHR services need to be a cornerstone in every healthcare system and be prioritised and supported by governments. With today’s changing political landscapes and conflicts in many parts of the world, this is more important than ever.

## Author contributors

KGD has been responsible for funding acquisition and principal investigator for the research projects together with JB. CF, ELT, AC, ECL, MKL, and KGD conceptualised the manuscript. CF, ELT, AC, ECL, MKL, JB, SA, HK, OK, and KGD organised and coordinated the mentioned workshops. CF and ELT performed the literature search and wrote the first draft of the manuscript. AC, MKL, ECL, and KGD edited the manuscript. AC, MKL, EL, KGD, SA, HK, OK, MT, AK, and AA provided scientific input and critically reviewed the manuscript. All authors agreed on the final version of the manuscript.
